# First case report of uterine perforation and bowel incarceration following a clandestine abortion in Morocco

**DOI:** 10.1093/jscr/rjac235

**Published:** 2022-05-31

**Authors:** Tarik Deflaoui, Rachid Jabi, Anas Derkaoui, Abdelali Merhoum, Yassin Kradi, Mohammed Bouziane

**Affiliations:** 1 Department of General Surgery, Mohammed VI University Hospital, Oujda, Morocco; 2 Laboratory of Anatomy, Microsurgery and Surgery Experimental and Medical Simulation (LAMCESM), Faculty of Medicine and Pharmacy, Mohammed I^st^ University, Oujda, Morocco

## Abstract

Uterine perforation is a rare complication of abortion. It becomes much rarer when associated with a small bowel incarceration at the uterine breach. Its diagnosis can be suspected clinically, but radiology remains more sensitive for diagnosis. Surgery is the cornerstone in treating this entity as it provides both diagnostic and therapeutic management. A multidisciplinary therapeutic approach should be immediately performed to ensure a good prognosis. In this report, we describe a case of small intestine incarceration in the breach of a uterine perforation that occurred in the weeks following a clandestine abortion.

## INTRODUCTION

Voluntary abortion performed in medical facilities is currently legalized in several Western countries [1]. However, it is still performed by harmful methods in other countries such low- and middle-income countries [2]. Hence, these unsafe procedure may be associated with serious complications such as uterine perforation, despite rare [3] and much rarer the small bowel incarceration at the uterine breach. In this report, we describe a case of a Moroccan woman who presented uterine perforation and bowel incarceration 10 days after a clandestine abortion by inserting of a foreign tool. To the best of our knowledge, this is the first case to be reported on this rare entity in our setting.

## CASE REPORT

**Figure 1:**
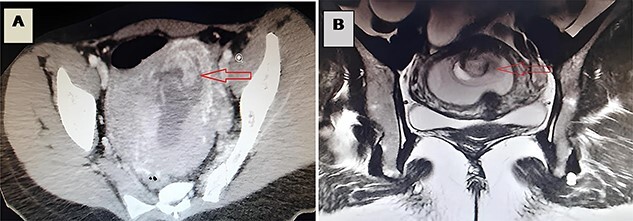
(**A**) A CT image revealing incarceration of a loop through a defect in the posterior wall of the uterus; (**B**) an MRI image revealing incarceration of a loop through a defect in the posterior wall of the uterus.

A 29-year-old woman, gravida 1 para 1 (vaginal delivery, 1 child died on the 20th day of life), presented to the emergency department with abdominal pain, nausea and vomiting developed during 5 weeks. She had not passed flatus for 1 week. These symptoms began 1 week after an evacuation of a 6-week pregnancy. Her past medical history was unremarkable. On initial physical examination, the patient was conscious, hypotensive at 90/50 mmHg, tachycardic at 110 p/m, eupneic at 16 c/m, SaO2 95% on room air and apyretic at 37°C.The abdominal examination showed an abdominal distention and tenderness, with diffuse tympany but without any detectable masses. A long-closed posterior cervix was noted with curdled milky secretions on vaginal manual examination. The use of a speculum revealed a normal-looking cervix with a delivered polyp through the cervix with curdled milky leucorrhea. Biological assessment using blood count found a hyperleukocytosis (15 220/μl) with a normal C-reactive protein and an elevated human chorionic gonadotropin (100 000 UI/l). The pelvic ultrasonography revealed an intrauterine ovular sac, a tubular material in the uterine cavity suggesting an incarcerated bowel. A complementary abdominal and pelvic computed tomography (CT) scan was performed (Fig. 1A, Supplementary Video 1), completed by an magnetic resonance imaging (MRI) (Fig. 1B, Supplementary Video 2). They showed a small intestine obstruction due to incarceration of an intestinal segment through a defect in the posterior wall of the uterus, a spontaneously hyperdense uterus and an intra-mural extravasation of the contrast material.

**Figure 2:**
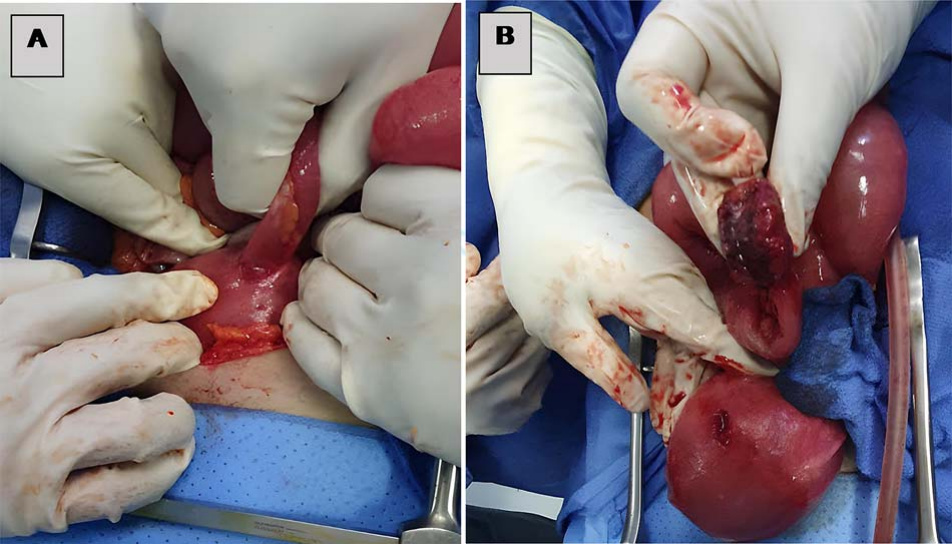
(**A**) Small bowel incarcerated at a uterine fundal perforation and (**B**) uterine perforation after loop extraction.

**Figure 3:**
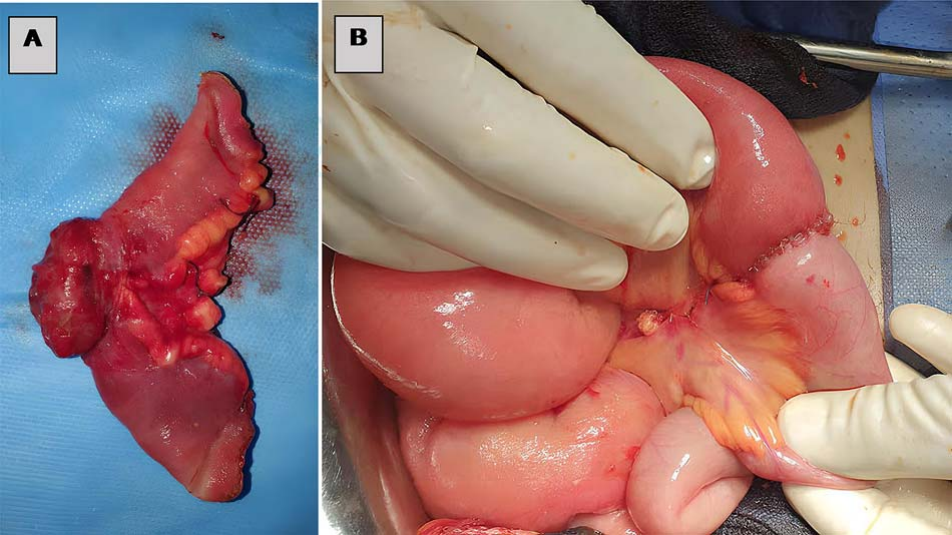
(**A**) Resected small bowel segment, (**B**) intestinal anastomosis.

**Figure 4:**
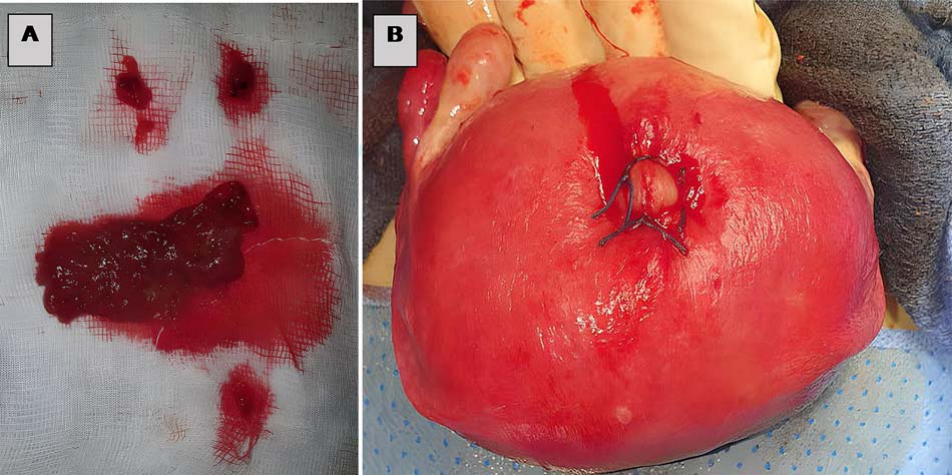
(**A**) Trophoblastic tissue, (**B**) closure of the uterine breach by X-stitches.

The patient underwent an exploratory laparotomy, which confirmed the obstructive etiology with a segment of the small bowel that was found incarcerated in a uterine fundal perforation (Figs 2A and B, Supplementary Video 3). The bowel, which was reduced in the abdomen, did not seem to be viable. A perforation of 1 cm was detected on the trapped surface and was lengthwise resected for 7 cm (Fig. 3A) treated by an intestine anastomosis (Fig. 3B). A uterine revision with an aspiration of trophoblastic tissue was also performed (Fig. 4A) and a closure of the uterine breach was performed by X-stitches (Fig. 4B). The postoperative period was uneventful.

## DISCUSSION

Although elective abortion is considered as a safe procedure, complications can be serious. Several reports showed worrying results, as 45% of abortions were considered risky [2]. In 2012, developing countries alone had 7 million women hospitalized per year for complications of unsafe abortion. Among the complications of this type of procedure is uterine perforation [4], which is a rare complication (0.05–1.9%) but of serious consequences [5]. Several risk factors of uterine perforation during abortion have been identified including surgeon’s experience as the most potent influencing factor [6] in addition to advanced age of woman, multiparity, history of previous abortions or C-sections, non-use of ultrasonography, inaccurate term of the pregnancy and when the procedure is performed by using non-medical devices [7, 8]. Bowel incarceration is a very rare complication of uterine perforation. It can be recognized clinically by an abdominal pain, distention, vomiting and nausea associated with gynecological signs that follow an abortion [5]. Imaging is key in the diagnosis of an incarcerated bowel in a uterine perforation. The diagnosis of an incarcerated bowel in a uterine perforation was reported for the first time using a CT scan by Dignac *et al*. in 2008 [9]. CT imaging is superior for primary diagnosis in these cases, especially with highly suspicious equivocal ultrasonographic findings.

Indeed, the uterine wall may interfere with the visualization of the intrauterine bowel, but the fatty nature of the bowel mesentery can be well detected on CT, which was historically highlighted by Dignac *et al*., a sign that should be considered as an alarm signal of bowel incarcerated in a uterine perforation [9]. In our patient, intestinal perforation could happen during the aggressive clandestine curettage procedures. Therefore, the therapeutic management is based on the removal of the bowel from the uterus and its subsequent reduction in the abdomen [10]. Sometimes large portions of the bowel may require resection with or without diversion [11, 12], followed by repair of the uterine breach, and sometimes hysterectomy can be indicated if the uterus is considerably damaged [12].

## CONCLUSIONS

The bowel incarceration following a uterine perforation is a very rare and harmful complication of non-medical abortions. The clinical manifestations are associated with small bowel obstruction and gynecological signs. Imaging has a considerable role for the diagnosis but laparoscopic exploration is recommended for both treatment and exploration.

## CONSENT

A written consent was provided by the patient to publish his data.

## CONFLICT OF INTEREST STATEMENT

None declared.

## FUNDING

None.

## Supplementary Material

Sup_Video_1_rjac235

Sup_Video_2_rjac235

Sup_Video_3_rjac235
